# Supplementary material for: Family caregivers’ perspectives on the acceptability of four interventions proposed for rural transitional care: A multi-method study
Source: PLoS One. 2022 Dec 19;17(12):e0279187. doi: 10.1371/journal.pone.0279187 (PMC9762580; doi:10.1371/journal.pone.0279187)
Supplement: S1 File — (DOCX) [file pone.0279187.s001.docx]

**Supporting Information**

**Intervention Lay Summaries**

**Introduction to lay summaries.** The lay summaries describe 4 interventions. An intervention is a healthcare practice or service. The 4 interventions were developed based on research. They all start in the hospital. The first one is discharge planning; it lays the foundation for the other 3 interventions.

**Lay Summary: Discharge Planning**

**Goals:**

1. Prepare patient and family (e.g. spouse, children, friends) to manage care and recovery at home after a hospital stay.
2. Make sure the patient’s care needs continue to be met after they go back home.
3. Make sure the patient’s care is coordinated as they go back home.

**Who will receive this intervention?** Patients in hospital who are being discharged and family who are providing them with care/support.

**Who will provide this intervention?** Nurses in collaboration with the healthcare team in hospital and at home.

**What does this intervention involve?**

In hospital, starting within 24 hours of admission, the nurse:

**- Assesses what the patient and family need to be able to manage care** and recovery **at home (e.g. Do they need to learn how to manage the patient’s health condition or use the treatments? Does the patient need to be able to walk up stairs?)**

**- Assesses the patient’s risk of being readmitted to hospital after discharge.**

- Asks the patient and family about their goals, preferences, and what they think they will need to be able to manage care and recovery at home.

During hospital stay, the healthcare team:

- Es**timates how long the patient will stay in the hospital.**

**- Involves the p**atient and family in creating a care plan to help them reach their goals.

- Discusses the supports needed to manage care and recovery at home.

- Discusses the level of functioning that the patient needs to be able to manage care and recovery at home.

- Comes to an agreement with the patient and family on what they need to manage care and recovery at home (e.g. they need to know how to use oxygen to manage patient’s trouble breathing at home).

- Comes up with strategies to address the needs and goals (e.g. order in-home oxygen; teach patient and family how to use it to manage trouble breathing; arrange for home care nurse to follow-up at home).

- Teaches the patient and family how to manage the patient’s condition at home (e.g. manage difficulty breathing due to lung disease), treatments (e.g. medications, oxygen therapy), and signs that the condition is getting worse (e.g. chest pain that doesn’t go away with heart medication). Asks the patient and family to repeat this information back to make sure they understood it. Clarifies anything that was not understood.

The nurse reviews the patient’s condition every day and whether they are reaching their goals. If there are any barriers to reaching the goals, the nurse lets the team know. The team, patient and family then change the care plan to help the patient and family meet their goals.

In hospital, within 24 hours before discharge, the nurse:

- Informs community healthcare providers (e.g. family doctor, home care nurse) of the patient’s discharge and care plan.

- Gives the patient and family written information that is easy to understand. The information **includes** instructions about treatments (e.g. **medications), devices (e.g. walker), equipment (e.g. grab bars in bathroom), diet, pain management, and physical activity;** what to expect at home; **problems to watch for or potential warning signs that indicate the patient’s health condition is getting worse; follow-up appointments; any home care arrangements that have been made; and c**ontact information.

- Teaches the patient and family how to manage the patient’s condition at home (e.g. manage difficulty breathing due to lung disease), treatments (e.g. medications, oxygen therapy), and signs that the condition is getting worse (e.g. chest pain that doesn’t go away with heart medication). Asks the patient and family to repeat this information back to make sure they understood it. Clarifies anything that was not understood.

**What benefits are expected?** Hospital and home care meets the patient’s and family’s needs and preferences. They have the knowledge, skills, and resources (e.g. home care services) they need to manage the patient’s care and recovery at home. The patient’s health condition will be managed successfully. Reduced complications, emergency room visits and hospital re-admissions.

**Lay Summary: Treatments**

**Goals:**

1) Make sure that patients and families (e.g. spouse, children, friends) know about the treatments (e.g. medications, special diets, oxygen therapy, wound or incision care) they need to use at home after discharge.

2) Increase patient’s and family’s confidence and ability to use treatments correctly.

3) Improve patient’s health.

**Who will receive this intervention?** Patients being discharged from hospital and family who are providing them with care/support after discharge from hospital.

**Who will give this intervention?** Nurses in hospital and at home in collaboration with the healthcare team. Patients and family at home.

**What does the intervention involve?**

In hospital, within 24 hours of admission:

-The nurse assesses patient’s and family’s ability to use treatments at home and **what** they **need to learn.**

**-The patient and family tell the healthcare team all the patient’s medications** (prescribed, over the counter, vitamins, supplements) used at home.

- The healthcare team makes sure all medications used at home that need to be continued in hospital are continued.

During hospital stay

The patient and family identify their goals around learning to use their treatments and help develop a care plan to meet the goals**.**

The nurse:

- Asks patient and family about barriers (e.g. lack of a system to organize medications, lack of money, lack of knowledge) that are preventing them from getting or using the treatments as prescribed.

- Helps patient and family to problem solve or follows-up with the right healthcare team member (e.g. pharmacist for a system or pill box to organize medications; social worker for financial assistance; occupational therapist for assistive devices).

- Teaches the patient and family what the treatments are for and how to use them.

- Gives the patient and family written information on the treatments that is easy to understand. **The information includes: the name of the treatments, how they work, how much to use, when and how to use them, how long to use them for, where to get them, and possible side effects.**

The patient and family explain this information back to the nurse to make sure they understood it. They show nurse how they use the treatments.

The nurse clarifies anything that was not understood and asks the patient and family to explain it or show how they use the treatments again.

In hospital, within 24 hours before discharge

The healthcare team makes sure that all treatments that need to be continued at home are prescribed.

The nurse:

- Informs the patient and family of the treatments and describes what they are for, their benefits and side effects, and how to use them.

- Explains how the new treatments relate to the old ones.

- Shows how to use the treatments and any related equipment.

- Makes sure they have this information in writing in language that is easy to understand.

The patient and family explain this information back to the nurse to make sure they understood it. They show the nurse how they use the treatments.

The nurse clarifies anything that was not understood and asks the patient and family to explain it back or show how they use the treatments. Provides feedback on any misunderstood or missed steps in using the treatments.

At home, 24 – 48 hours after discharge, the nurse:

- Visits the patient and family at home to review discharge instructions on treatments, assess patient’s health condition and ask how things are going with the treatments.

- Makes sure the treatments are the right ones.

- Asks the patient and family about any questions or problems they have with the treatments.

- Asks about all treatments being used including over the counter medications, supplements and remedies.

- Asks about treatments that were prescribed but are not yet obtained or being used.

- Asks about barriers to getting or using the treatments. Helps to problem solve. Problem solving may include: discussing the possible risks of not using the treatment; or contacting the family doctor or pharmacist to check the treatment or encouraging patient or family to do so.

Patient and family explain each treatment, what it is for, how they are taken, etc and show how they use each treatment.

The nurse clarifies anything that was not understood and asks the patient and family to explain it and show how they use the treatments. Provides feedback on any misunderstood or missed steps in using the treatments.

Month following discharge, the nurse:

- Contacts the patient and family by phone during any week a home visit is not done.

- Asks how they are managing and if they have any questions about treatments.

- Reviews treatments and if there have been any changes prescribed and if all prescribed treatments are being used.

- Asks about problems in getting or using treatments and tries to solve the problem. May contact primary healthcare provider (e.g. family doctor) if unable to solve the problem.

- Reinforces how important it is to continue the treatments and to contact the appropriate healthcare provider for help if needed.

- Assesses patient’s and family’s need for continued support in managing treatments.

**What benefits expected?** Patients and families have the knowledge, skills, confidence and resources needed to use the treatments correctly. Improvement in the patient’s health (e.g. pain, breathing). Reduced complications, emergency room visits and hospital re-admissions.

**Lay Summary: Warning Signs**

**Goal:** Patients and families (e.g. spouse, children, friends) know the “warning signs” that may indicate health conditions are getting worse and what to do about them.

**Who will receive this intervention?** Patients being discharged from hospital and family who are providing them with care/support after discharge from hospital.

**Who will provide this intervention?** Nurses in collaboration with healthcare team in hospital and at home. Patients and families at home.

**What does this intervention involve?**

In hospital, within 24 hours of admission, the nurse assesses:

- What the patient and family already know about the warning signs and their ability to detect and respond to them at home.

- What they **need to learn so that they know what to do at home.**

During hospital stay, the nurse:

- Teaches the patient and family about the warning signs for the patient’s conditions (e.g. how to watch out for them and recognize them, when to get medical help, who to contact, and when to go to the emergency room).

- Asks the patient and family to explain this information back to make sure they understood it. Clarifies anything that was not understood and asks the patient and family to explain it back again.

- Gives them written information on the warning signs that is easy to understand.

In hospital, within 24 hours before discharge, the nurse:

- Reviews the warning signs again with the patient and family (e.g. how to watch for and detect them, when to get medical help, who to contact, when to go to emergency)

- Asks the patient and family to explain this information back to make sure they understood it. Clarifies anything that was not understood and asks the patient and family to explain it back again.

- Makes sure they have the list of warning signs to watch out for and what to do.

At home, 24 – 48 hours after discharge, the nurse:

- Visits the patient and family at home to assess patient’s health condition(s) and asks if they have any questions about the warning signs. Asks if they detected any and what they did. Validates their learning (e.g., lets them know the positive steps they took).

- Reviews the warning signs again with the patient and family (e.g., how to watch for and detect them, when to get medical help, and who to contact)

- Reinforces the importance of watching out for the warning signs and what to do if they are detected.

- Asks the patient and family to explain this information back to make sure they understood it. Clarifies anything that was not understood and asks the patient and family to explain it back again.

Month following discharge, the nurse:

- Contacts patient and family by phone any week a home visit is not done. Asks about patient’s condition, how they are doing watching out for warning signs, if they detected any and what they did about them. Validates the positive steps the patient & family took.

- Reviews warning signs, and when and who to call if any are detected.

- Reinforces the importance of continuing to watch out for the warning signs.

- Assesses the patient’s and family’s need for continued support.

**What benefits are expected?** Patients and families know which warning signs they need to watch for, how to watch for and detect them. They know what to do if they find any. They know when to go to the emergency room. Prevents health conditions from getting worse. Prevents complications. Fewer emergency room visits and hospital re-admissions.

**Lay Summary: Physical Activity**

**Goals:**

1) Make sure patient and family (e.g. spouse, children, friends) know the importance of physical activity (e.g. getting up out of bed) during recovery.

2) Promote safe physical activity during recovery.

3) Prevent declines in patient’s ability to do physical activities (e.g. ability to get up, ability to walk).

4) Promote return to usual daily activities.

**Who will receive this intervention?** Patients being discharged from hospital and family who are providing them with care/support after discharge from hospital.

**Who will deliver this intervention?** Nurses in collaboration with rehabilitation therapists and healthcare team in hospital and at home. Patients and family at home.

**What does this intervention involve?**

In hospital, within 24 hours of admission, the nurse:

- Asks about patient’s ability to do physical activities (e.g. can they walk on their own?).

- Assesses if patient has had any declines in their ability to do physical activities (e.g. can’t walk on own now but could 2 weeks ago). If detects any declines, involves rehabilitation or physical therapist.

- Asks if patient uses mobility devices (e.g. cane, walker) and arranges to get them.

During hospital stay**,**

Patient, family and healthcare team:

- Identify patient’s physical activity goals (e.g. get back to walking 4 km on own).

- Develop a care plan to meet these goals. The plan includes being up for a certain amount of time (e.g. at least 3 and 1/2 hours per day) and walking a certain amount (e.g. medical patients aged 65+ should walk at least 900 steps per day or 690 meters).

The nurse:

- Checks that the patient does not have medical restrictions to getting up.

- Assesses barriers to patient’s mobility. Tries to address the barriers. For example, if patient is afraid of falls, suggests walking with another person or walking on the spot at bedside. If catheters or sedatives are making it difficult to get up, discusses with the healthcare team if they can be stopped or changed. If pain, then recommends the patient take prescribed medication.

-Teaches the patient and family about the risks of bed rest (e.g. blood clots, dizziness, muscle loss) and need for physical activity during recovery.

- Teaches about safe mobility (e.g. wear non-skid shoes or slippers with closed heel and toe, remove trip hazards, put lights on, wear glasses and hearing aids).

- Teaches how to balance physical activity with rest (e.g. walk for a few minutes at first, take a rest, then walk again).

- Gets the patient up at least 3 times a day within 24 hours of hospital admission or surgery. May start by sitting on the edge of bed or in a chair. If patient feels dizzy, asks patient to do some simple exercises (e.g. lift heals up and down) and gives tips (e.g. keep sitting for a few more minutes and get up slowly) on how to help manage it. If patient feels OK, starts walking.

- Watches or helps the patient walk, depending on patient’s condition.

- Encourages patient to balance physical activity with rest but to not lie down in bed, a sofa, or reclining chair most of the day.

- Encourages gradually increasing walking and time spent up.

- Gives patient a pedometer or accelerometer and/or writing pad to write down the time spent out of bed, amount walked, and any barriers to getting up and walking.

The patient: Gradually increases amount of walking and time spent up daily depending on their condition, goals and care plan.

- Does this in addition to any rehabilitation or physical therapy they may be getting.

- Writes down every day the number of steps or distance walked, amount of time up, other physical activities done, and any barriers.

The family: gives encouragement and support (e.g. walks with patient, records amount of walking and time spent up, barriers to getting up).

In hospital, within 24 hours before discharge, the nurse and other members of the healthcare team:

- Go over patient’s physical activity goals with patient and family, how to achieve them by addressing the barriers and using strategies learned in hospital.

- Make sure patient has devices (e.g. walker) and equipment (e.g. grab bars installed at home). If not, makes sure they are ordered.

- Discuss with patient and family **the level of walking, amount of time to spend up, other physical activities, and any** prescribed exercises the patient needs to do at home. Make sure patient and family have written instructions.

- Make **sure that patient and family understand their purposes, when and how to do them, and how many times and how often to do them.**

At home, 24 - 48 hours after discharge, the nurse:

- Visits patient and family at home to go over recommended walking, amount of time to spend up, and any prescribed exercises.

- Goes over patient’s physical activity goals and progress made to date.

- Asks about any prescribed mobility devices (e.g. cane, walker) and equipment (e.g. grab bars) and if they got them and are using them.

- Asks about any problems or barriers they had.

- Helps patient and family problem solve.

- Assesses safety of home for walking.

- Goes over safe mobility strategies and how to balance physical activity and rest.

- Asks about future appointments with rehabilitation or physical therapist.

- Recommends looking for opportunities to walk (e.g. walking together to the kitchen for meals instead of bringing meals to the patient).

The patient: Records daily the amount of time spent up, the number of steps or distance walked, other activities done, and any barriers to walking and doing other activities.

- Increases the number of steps or distance walked daily and amount of time spent up, based on how they feel, their condition and goals. For example, medical patients aged 65 and older should walk at least 900 steps/day or 690 meters.

- Does any prescribed exercises.

Family gives encouragement and support (e.g. helps with recording).

Month following discharge, the nurse:

- Contacts patient and family by phone during any week that a home visit is not done.

- Reviews patient’s physical activity goals.

- Reminds about the importance of increasing walking and amount of time spent up based on patient’s health condition, care plan and goals.

- Suggests light activities to do around the house based on health condition and goals.

- Asks about average number of steps or distance walked every day and time spent up.

- Asks if any problems doing everyday activities and any prescribed exercises, using or getting adaptive devices and equipment. Helps to problem solve.

- Contacts relevant healthcare provider if unable to solve the problem or encourages patient or family to do so.

- Reinforces safety and other strategies (e.g. balancing physical activity and rest, getting up slowly if dizzy).

- Reminds patient about the importance of not spending most of day lying down, increasing amount of walking daily and being up, doing any prescribed exercises, and of contacting the appropriate healthcare provider as needed.

**What benefits are expected?** Patient and family know the risks of bed rest, the importance of physical activity, and how to address barriers that keep patient from being physically active during recovery. Patient is physically activity during recovery. Returns to usual level of physical activity and daily activities, as much as possible. Reduced complications (e.g. falls), emergency room visits and hospital re-admissions.
