# Supplementary material for: Family caregivers’ perspectives on the acceptability of four interventions proposed for rural transitional care: A multi-method study
Source: PLoS One. 2022 Dec 19;17(12):e0279187. doi: 10.1371/journal.pone.0279187 (PMC9762580; doi:10.1371/journal.pone.0279187)
Supplement: S2 File — (DOCX) [file pone.0279187.s002.docx]

**Supporting Information**

**Semi-structured Interview Guide for Families**

**Perspectives on transitional care experience to identify unmet needs**

1. Prompt the participants for more information about their post-discharge experience, guided by the following questions:
2. I would like to know about your experience since your relative came home from the hospital and some of the things that went well and some of the things you found challenging or hard to manage. I would like you to think back to your recent experience and to tell me during the first few weeks after leaving the hospital, were there any parts of your care and recovery at home that you found hard to manage?

[Probes for self-care/management areas:

- Treatments (e.g. new medications/special diets/oxygen therapy, wound or incision care). Were you able to manage your relative’s treatments? If not, why? Can you think of any things that would have made it easier for you to manage their treatments?

- Physical activity. Were you able to manage whatever physical activity (e.g. walking) was recommended for your relative after coming home? If not, why? Was there anything about the recommendations you found difficult to manage at home? Can you think of any things that would have made it easier for you to manage your relative’s physical activity and meet the goals that were set with the nurses and other healthcare providers?

- Warning signs (i.e. knowing signs that your relative’s health has worsened, and who to contact for medical professional help and advice). Were you able to recognize warning signs that your relative’s health might be getting worse and contact medical professionals for help and advice? If not, why? Can you think of any things that would have made it easier for you to recognize warning signs and seek help and advice?]

Were there any other things you felt unprepared for in managing your care and recovery at

home?

2) Tell me about the help that you received at the hospital to prepare you to manage your relative’s care and recovery at home? How did your relative’s healthcare team prepare you? Could the nurses and other healthcare providers have better prepared you to manage your relative’s care and recovery after returning home? If so, how?

[Probes for preparation for self-care/management:

- Did they ask if you had any concerns about managing your relative’s care and recovery at home after discharge from the hospital?
- Did they ask about what you would need to manage your relative’s care and recovery at home after discharge from the hospital?
- Did they tell you what to expect after your relative’s discharge from the hospital and what to do to manage your relative’s health and recovery at home? If so, how? (e.g. informal discussion, formal meeting, written material).
- In your opinion, what, if anything, could they have done better to prepare you to manage your relative’s care and recovery at home?]

3) Tell me about the help you received or have been receiving from healthcare providers since your relative came home from the hospital in managing their care and recovery at home.

[Probes for care from nurses and other healthcare providers since coming home:

- At any point since coming home, did you need more help in managing your relative’s care and recovery? (e.g. manage treatments; rest and physical activity; warning signs?)
- How could you have been better supported in managing your relative’s care and recovery at home?]

4) Summary of first part of interview and other needs: Based on what you have said so far, I am hearing that you are finding [name the problem in family’s own words] to be difficult and that you felt unprepared to manage. Is that right? Are there any other things you have found difficult and unprepared for now that your relative is back home that you think are important? Is there anything about your relative’s discharge or transitional care experience that we haven't touched on in that you think is important to address?

**Perspectives on the interventions**

As somebody who has gone through a transitional care experience in your rural community, let me know how you would assess this intervention based on that.

1. **Discussion of interventions rated unacceptable.** Based on your responses, I see that [*name intervention*] was not completely acceptable to you. Can you tell me why? What was it about this intervention that you did not find acceptable?

If not yet addressed by participant, ask about relevant scale items:

1. *Effectiveness*: You indicated that this intervention would not work/help. Can you tell me why? How could this intervention be changed to make it more helpful? [Probes: *content, activities, dose, format* *and* *mode of delivery].*
2. *Appropriateness:* You indicated that this intervention is not a good fit you. Can you tell me why? How could this intervention be changed to make it a better fit for you?

[Probes *content, activities, dose, format* *and* *mode of delivery].*

1. *Likelihood of use*: You indicated that you would be unlikely to use/participate in this intervention. Can you tell me why? In your opinion, what would prevent you from using it? How could this intervention be changed to make you more likely to use it?

[Probes *content, activities, dose, format* *and* *mode of delivery].*

1. *Risks*: You indicated that there would be risks associated with participating in this intervention. Can you tell me more about this? What are the risks that you fear? How could this intervention be changed to reduce those risks? [Probes *content, activities, dose, format* *and* *mode of delivery].*

Is there anything else important to know about this intervention and why it would or would not meet your needs? [Probes: burden, self-efficacy/confidence, costs (profits of values that must be given up to engage in the intervention)].

1. **Intervention interview wrap-up** **question**. Are there any other interventions that we haven’t talked that you think would help you to manage your relative’s care and recovery at home?

*Note*. Italicized text and text in brackets refer to our conceptualization or that of the Intervention Perception Scale and will not be used with the participants.
